# Genomic insights into runs of homozygosity, effective population size and selection signatures in Iranian meat and dairy sheep breeds

**DOI:** 10.1371/journal.pone.0323328

**Published:** 2025-06-11

**Authors:** Zohre Yousefi, Mohammad Hossein Moradi, Mohammad Taghi Beige-Nasiri, Masoud Shirali, Rostam Abdollahi-Arpanahi

**Affiliations:** 1 Department of Animal Science, Agricultural Science and Natural Resources University of Khuzestan, Ahvaz, Iran; 2 Department of Animal Science, Faculty of Agricultural and Natural Resources, Arak University, Arak, Iran; 3 Department of Animal Science, University of Tehran, Karaj, Iran; 4 Agri-Food and Biosciences Institute, Hillsborough, United Kingdom; 5 School of Biological Sciences, Queen’s University Belfast, Belfast, United Kingdom; National Bureau of Animal Genetic Resources, INDIA

## Abstract

Genome-wide scan for run of homozygosity (ROH) stretches, effective population size (N_e_) and selection signatures can help to elucidate mechanisms of selection and pinpoint genomic regions linked with phenotypic traits. This study aimed to identify the genomic patterns of ROH, N_e_ and selection signatures in two Iranian main sheep breeds including Afshari and Qezel (known as meat and dairy sheep, respectively) using 49,017 single nucleotide polymorphisms (SNPs) generated using the ovine 50K SNP BeadChips. Analysis of ROH in Iranian sheep breeds revealed the differences in the pattern of ROH length and burden in these breeds. Inbreeding estimated based on ROH stretches showed very low amount of inbreeding in these indigenous sheep breeds. The Qezel breed displayed a higher N_e_ than Afshari breed. Furthermore, the potential selection was detected in genomic regions using three complementary approaches including F_ST_ (fixation index), XP-EHH (cross-population extended haplotype homozygosity), and hapFLK (haplotype differentiation). Our results identified the genomic regions that were enriched with the genes associated with immune response (e.g., *IL23A, STAT2* and *DOCK5*), milk traits (e.g., *PCCA*, *ACAP3*, *TTK* and *BTG3*), energy metabolisms (e.g., *GLS2*), reproduction (e.g., *ANGPT2*), fecundity (e.g., *BMP5*), nervous system (e.g., *DLG2*, *PCDH9*, and *FRMPD4*), growth traits and muscle formation (*NPY*, *MYF5* and *PPP1R12A*), and sweat gland development (*SCNN1D*). Some regions were also detected for the first time and overlapped with no genes suggesting novel loci associated with traits that differentiate these breeds. Overall, the finding of this study may shed light on the genomic regions linked to economically important traits in sheep as well as for developing the conservation and selection breeding programs.

## Introduction

Locally adapted breeds are valuable resources for identifying genomic regions associated with adaptive and economically important traits. Archaeological and genetic evidence suggests that sheep were domesticated approximately 9000 years ago in a region west of Zagros Mountains of Iran and Iraq [1]. The diverse climatic conditions in Iran have contributed to the development of a wide variety of sheep breeds, each adapted to specific environmental challenges. However, the increasing prevalence of genomic inbreeding in these locally adapted breeds poses a significant threat to their genetic diversity. Over time, this reduction in genetic diversity may limit their ability to adapt to new breeding objectives and environmental changes, particularly in the context of ongoing climate change [2].

Genomic regions subjected to selection may exhibit high levels of homozygosity, also known as runs of homozygosity (ROH). ROH segments are defined as consecutive stretches of homozygous genotypes that are present in an individual as a result of transmitting identical haplotypes from parents to their progenies [3]. Some studies have also used the extent of ROH across the genome as a measure of inbreeding [4–6]. ROH detection is widely applied to investigate the level of selection pressure on the populations [7]. The ROH length and frequency can reveal details about the genomic regions that have experienced recent or past selective pressure [8]. Furthermore, effective population size (N_e_) is another important parameter for the evaluation of genetic variation within a livestock population and its evolution across time. The population structure analysis based on linkage disequilibrium (LD) might offer an alternative view for the estimation of N_e_ when the pedigree information is not accessible [9].

The domestication of animals and the natural and artificial selection afterward, for either morphological or economical traits, leave some detectable footprints on the genomes. Over thousands of years of selections, different sheep breeds have evolved all around the world displaying a wide range of phenotypic traits [10]. The establishment of ovine genome-wide SNP panels enables the identification of genomic regions undergoing selection, linked to various traits, to enhance comprehension of evolutionary mechanisms [11].

Several approaches including Wright’s fixation index (F_ST_), cross-population extended haplotype homozygosity (XP-EHH), and haplotype differentiation (hapFLK) have been used to identify the footprints of selection. The F_ST_-approach is based on the measure of population differentiation owing to allele frequencies of single locus between populations [1,12,13]. Haplotype differentiation and LD patterns contain useful information to examine selective sweeps or patterns of diversity. Sabeti et al. [14] proposed the XP-EHH method which assesses haplotype differences between two populations. The XP-EHH approach compares haplotype lengths between populations to examine local variation in the recombination rates and to detect selected alleles [14]. Most of the methods based on haplotype differentiation fail to consider the potential presence of hierarchical population structure, leading to an increased risk of both false positives and negatives errors [15]. Hence, the approach of hapFLK suggested by Fariello et al. [15] is a haplotype-based extension of FLK method. This methodology considers the heterogeneity in population size and the hierarchical relationships among populations. We expect the combined use of above approaches could alleviate the limitation of the single method to a certain extent. These methods successfully reported the putative genomic regions and the candidate genes associated with divergent traits including body size and skeletal morphology (e.g., Kim et al. [16]), reproductive performance (e.g., Taye et al. [17]), nematode resistance (e.g., McRae et al. [13]), fat deposition (e.g., Moradi et al. [1]), coat color (e.g., Fariello et al. [18]), immune response (e.g., Onzima et al. [2]) and muscle formation (e.g., Purfield et al. [19]).

**Fig 1:**
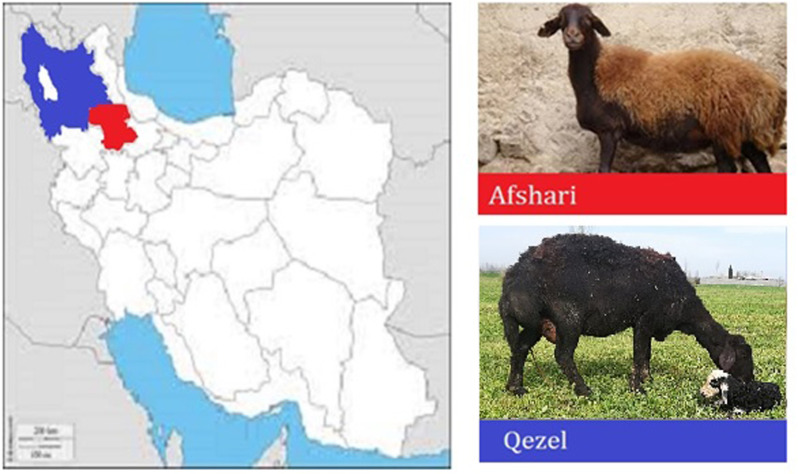
Geographic origin of the breeds used in the current study in Iran. Afshari and Qezel breeds are marked with red and blue respectively.

In the present study, two important sheep breeds of Iran namely Afshari and Qezel have been studied. The interesting point regarding these two breeds is that both are located and reared in a very close geographical area (neighbor provinces) with very similar morphological characteristics in terms of color and shape, but are classified and reared for different production traits (Fig. 1). Afshari sheep is one of the heaviest Iranian sheep breeds that is mostly known as a meat sheep which has high potentials for fattening, reproduction (litter size) and adapting in the cold climate [20]. The Qezel sheep originates from northwestern regions of Iran and northeastern Turkey, particularly in the areas known as Azarbayjan, which has harsh climatic condition of dryness and coldness in its mountainous terrain. The wool of this sheep displays a range of colors from light brown to dark brown, with the wool on its legs typically being of a darker hue. The Qezel are used for both wool and milk in this region, although, is mostly known as a dairy sheep breed in Iran, rearing for producing a special kind of white cheese namely Lighvan [21].

This study aimed to measure the occurrence and distribution of ROH in different lengths, ROH-based genomic inbreeding and N_e_ in two Iranian sheep breeds, as well as to identify the genomic regions under positive selection The results of the present study would be important for identifying the candidate regions or the genes of interest among breeds that help understanding the evolutionary and biological mechanisms involved in the phenotypic manifestation.

## Materials and methods

### Ethics statement

All methods and animal care and handling procedures were allowed and approved by the Arak University Animal Care and Use Committee (No. 2014/12534). All efforts were carried out in accordance with relevant regulations to minimize any discomfort during blood collection. Since the genotypic data used in this study were obtained from the previously published study [10], no additional owner consent was required. The authors also complied with the ARRIVE (Animal Research: Reporting of In Vivo Experiments) guidelines.

### Animals and genotype quality control

The genotypic data of two Iranian sheep breeds consisting Afshari (41 heads) and Qezel (35 heads) were used in the present study. The details of animal sampling have been described previously [10]. Briefly, the animals were selected randomly to ensure they were unrelated and representative of the population. Typically, 4–5 animals were sampled from each flock, covering different age classes to minimize genetic bias and provide a robust dataset for analysis [1]. Blood samples from Afshari sheep were collected from Zanjan province, while samples from Qezel sheep were obtained from East Azerbaijan province, Iran. Fig 1 depicted the geographic distribution of these breeds. These samples were genotyped using Illumina OvineSNP50 genotype panel.

Genotype quality control (QC) was conducted using PLINK v1.90 [22]. A number of QC measures were implemented to all SNPs as follows: Individuals and SNPs with a call rate <95% were discarded, and then the non-autosomal and the SNPs with a MAF < 0.02 across all individuals were excluded from further analysis [1]. The Hardy-Weinberg equilibrium test was conducted for the remaining SNPs in each breed. Any SNPs that were not at equilibrium in at least one population (p-value<10^-6^) were eliminated as genotyping errors [23]. To ensure that the removal of SNPs not in Hardy-Weinberg equilibrium did not disproportionately affect specific genomic regions, we examined the distribution of discarded SNPs and repeated our analyses with and without these SNPs. The discarded SNPs were widely scattered across chromosomes and their inclusion did not alter our results, confirming the robustness of our findings.

The SNP positions were mapped to the ovine genome assembly version 3.1 using the data from the Sheep HapMap dataset (http://www.sheephapmap.org) and eventually, the SNP markers with unknown chromosomal location were discarded. After QC, the final dataset used in this study was composed of 72 animals from two sheep breeds and has been described in Supplementary materials (S1, S2, S3 Tables). Finally, the missing genotypes imputation was conducted for each chromosome using Beagle [24].

### Population structure analyses (PCA)

Due to the potential for uncontrolled crossbreeding in the studied populations, which is common in locally adapted breeds, PCA was conducted using all genotyped markers to elucidate the assignment of animals to groups and to determine the population structure of the breeds under study. This analysis was performed using the GenABEL package in R software, version 3.4.1 [25].

### Runs of homozygosity (ROH) and genomic inbreeding (F_ROH_)

The ROH stretches were detected in each of the two populations using PLINK v1.09 [22] with sliding windows of 1000 kb across the genome [4]. The following principles were used to define the ROH: (i) a maximum of two missing SNPs and up to one heterozygous genotype were allowed within the ROH region, (ii) the minimum number of SNPs required to constitute an ROH was set at 40, (iii) the minimum SNP density within an ROH was one SNP per 100 kb, and (iv) the maximum allowable gap between two consecutive homozygous SNPs was 250 kb [4]. The minimum number of SNPs required to create a ROH (l) was estimated as Lencz et al. [26]:


$$l=\;\frac{\log_{e}\;\frac{\alpha }{n_{s}.n_{i}}}{\log_{e}\;\left(1-\overset{―}{het}\right)}\;$$

Where, *n*_*s*_ was the number of genotyped SNPs per individual, *n*_*i*_ was the number of individuals, α was the percentage of false positive ROH (set to 0.05), $\overset{―}{het}$ was the mean SNP heterozygosity across all SNPs. ROHs were identified for each individual separately. Each ROH was categorized according to its physical length into 1–5 Mb, 5–10 Mb, 10–15 Mb, 15–20 Mb, and ≥ 20 Mb. For each of the aforementioned ROH length classes, the mean sum of ROHs was calculated by summing the length of all ROH for each individual within each ROH class and then the results were averaged per breed [27].

The ROH based inbreeding coefficient (F_ROH_) was calculated by dividing the total length of all ROHs per animal by the total autosomal SNP coverage [28]:


$$F_{ROH\;=\;}\frac{{(L}_{ROH}}{L_{AUTO}})$$

In which, L_ROH_ is the total length of ROH per animal and L_AUTO_ denotes the total length of autosome covered by the SNPs (2.463 Gb).

### Effective population size (N_e_)

The past and current N_e_ for each breed was computed using the SNeP v1.1 tool as presented by Barbato et al. [29]. This method infer the historical and current N_e_ in the presence of mutation [30]:


$$N_{T(t)\;}=\;{\left(4f(ct)\right)}^{-1\;}\left({E\left[r_{adj}^{2}|ct\;\right]}^{-1}-\alpha \right)$$

Where, *N*_*T*(t)_ is the N_e_ at *t* generations ago calculated as *t* = (2f(c_t_))−1 [18], *c*_t_ is the recombination rate for a given physical distance between SNPs obtained using Sved and Feldman, (1973), *r*^2^_adj_ is the value of LD corrected for sample size and *α* is an adjustment for the occurrence of mutations. Only the SNPs with a MAF greater than 0.05 were employed to estimate the N_e_ (Supplementary Material, S1 Table).

### Determination of selection signatures

Three complementary approaches including F_ST_ [31], XP-EHH [14] and hapFLK [15] were calculated as described below

#### *F*_*ST*_.

To detect population-specific loci under positive selection, the F_ST_ value per each SNP was calculated using the R v 4.0.2, following the unbiased estimator suggested by Weir and Cockerham, [31]. A kernel regression smoothing algorithm [32] was applied to individual F_ST_ values, to facilitate the identification of genomic regions containing more extreme F_ST_ values using *Lokern* package in R v 4.0.2 [33]. The F_ST_ values for each set of 5 adjacent SNPs, were averaged and termed windowed F_ST_ as suggested by Moradi et al. [1]. The windowed F_ST_ values were then plotted against the genomic location and only the highest 0.1% of F_ST_ values were considered as signatures of selection [34].

#### XP-EHH.

To calculate XP-EHH, the ancestral allele for the ovine chip SNPs were obtained from the International Sheep Genomics Consortium (https://www.sheephapmap.org). Haplotypes phases were then reconstructed for each animal using fastPHASE version 1.2.3 [35]. The XP-EHH test was performed following the scripts in Pritchard lab website (http://hgdp.uchicago.edu/Software/). To account for genome-wide variations in haplotype length between populations, the XP-EHH scores were standardized by subtracting the mean and dividing by the standard deviation of all XP-EHH scores (for more details see Sabeti et al. [14]; Pickrell et al. [36]). The highest 0.1% of standardized XP-EHH scores were finally considered as signatures of selection for each population [34].

#### hapFLK.

This method was proposed by Fariello et al. [15] and it considers the haplotype structure of the population. The hapFLK score is calculated based on the difference in haplotype frequencies between populations using fastPHASE 1.4.0. This approach can take migration and demographic bottlenecks into account [15]. Reynolds distances and a kinship matrix [37] were computed by the hapFLK program, accessible at https://forg-edga.jouy.inra.fr/projects/hapflk/files. In this study, no outgroups were defined, 2 clusters (−K, 2) were applied and the hapFLK statistic was calculated for 20 run of expectation maximization (EM) algorithm to fit the LD model (–nfit = 20). The detailed description of this method can be found in Brito et al. [38]. The normalization of hapFLK values for each SNP on the genome was performed using the python scripts available in the hapFLK webpage (https://forge-dga.jouy.inra.fr/projects/hapflk), with a q-value threshold of 0.01 to minimize false positives [2]

### Bioinformatics analyses

We used the Ensembl database (https://www.ensembl.org/biomart) with the Biomart tool [39] on the sheep genome assembly 3.1 to identify the genes that were reported within the genomic regions of interest in Afshari and Qezel sheep. The genes within a 500 kb flanking distance from each region of interest were obtained and combined for functional analysis following previous studies in sheep [1] and Holstein dairy cattle [40].

All identified genes from the F_ST_, XP-EHH, and hapFLK methods were processed separately using the functional annotation tool implemented in DAVID tools 6.7 [41] to identify enriched functional terms. Since each method captures distinct aspects of selection pressure such as ancient selection, recent selection, and population differentiation, the results of each method were analyzed independently to ensure a comprehensive exploration of selection signatures. This approach leverages the unique strengths of each method. Overlapping selection signatures were defined as genomic regions (or genes within those regions) identified by at least two of the three selection signal methods (F_ST_, XP-EHH, and hapFLK). These regions were considered overlapping if they shared the same or closely adjacent chromosomal positions (within a 500 kb flanking distance).

## Results and discussion

### Data mining and population structure

Following quality control, 44152 and 44824 autosomal SNPs remained in the Afshari and Qezel breeds, respectively (Supplementary Material, S1 Table). The quality control steps varied depending on the statistical method used. Results for each method are described in the Supplementary Materials (S2 and S3 Tables).

**Fig 2:**
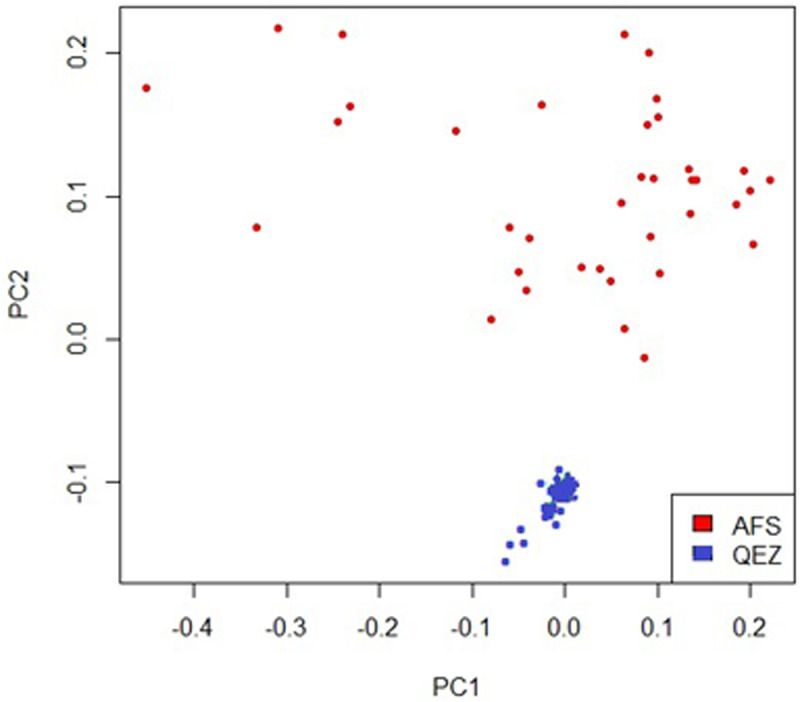
Principal component analysis results based on whole genome SNP data. Individuals are plotted according to their coordinates on of first principal component (PC1) versus second principal component (PC2), which explain 4.9% and 2.9% of total variance, respectively. The red and blue colors are used to show Afshari and Qezel breed animals, respectively.

Fig 2 depicts the scatterplot of the first two principal components (PC1 and PC2). The PCA results revealed that all animals were assigned to their respective breed groups, with clear separation between animals of two breeds along PC_2_ (Fig 2). The first and second components accounted for 4.9% and 2.9% of total variation in this analysis, respectively. The greater spread of Afshari individuals along PC2 suggests higher genetic diversity within this breed, likely due to its widespread use for crossbreeding and fattening across Iran, which may have introduced additional genetic variation.

PCA is a powerful technique for extracting axes with maximal variation from genomic data sets and is particularly useful for clustering analysis when clusters are not well defined, so animals of the same breed being positioned close together on PCA plots [42]. In this study, PCA was employed to assess population structure and genetic relationships, as it was assumed that some animals might not be purebred and uncontrolled crossbreeding could have occurred. The clear separation of the two breeds along PC2 highlights their distinct genetic backgrounds, while the spread of Afshari individuals along this axis underscores the impact of recent breeding practices on genetic diversity. Overall, our findings demonstrate that PCA successfully captured the genetic differentiation between the Afshari and Qezel breeds, providing valuable insights into their population structure.

### Runs of homozygosity (ROH)

**Fig 3:**
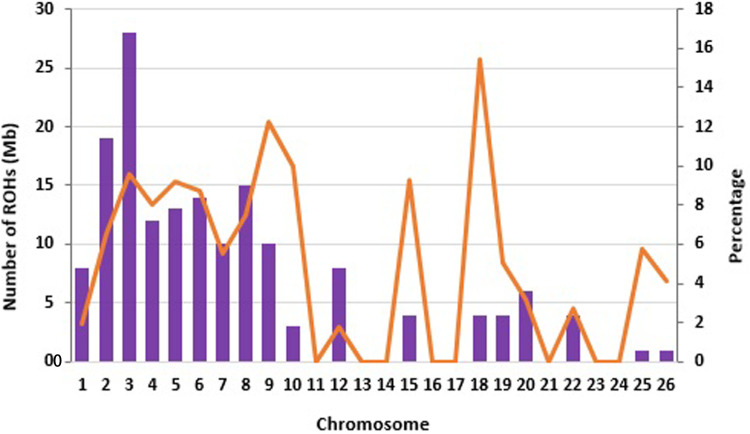
Distribution of the runs of homozygosity (ROH) across the chromosomes in two sheep breeds. The bars display the total number of ROH per chromosome identified in the animals that had at least one ROH. The orange line represents the average percentage (%) of each chromosome covered by ROH.

There were different numbers of ROH in the two populations. Afshari had a higher number of ROH (91) than Qezel (73). Chromosome 3 (OAR3) had the largest number of ROH and on average 70% (18 chromosomes out of all 26 autosomes) of the chromosome consisting of ROH (Fig 3). OAR18 and OAR9 had the largest percentage of the genome that were observed to be covered by ROH, with 15.41 and 12.23%, respectively (Fig 3). The average length of ROH was 5.48 Mb and the longest segment across breeds found on chromosome OAR3 was 82.76 Mb, which was composed of 1736 SNPs, followed by OAR5 with 73.10 Mb and 1533 SNPs. The ROH with smallest length was found in OAR26 (3.9 Mb) having 80 SNPs. In general, the total number of ROHs per chromosome decreases as the chromosome length shortens. In this data, no ROH was found on OAR11, 13, 14, 16, 17, 21, 23, and 24.

Abied et al. [5] investigated the genomic regions with the highest ROH frequencies within 96 sheep samples from five local Chinese sheep breeds. They reported that the highest percentage of ROH per chromosome were observed on chromosome OAR2 (11.39%) and OAR3 (11.31%). Al-Mamun et al. [27] in another study on five populations of Australian domesticated sheep reported that the largest proportion of ROH was obtained for OAR25 and OAR22 with 16.48 and 15.05%, respectively. Different chromosomes have been reported harboring the largest and the lowest proportion of ROH in worldwide sheep breeds [6,34] and it seems this depends on the breed of interest and the evolutionary process they experienced [19].

**Fig 4:**
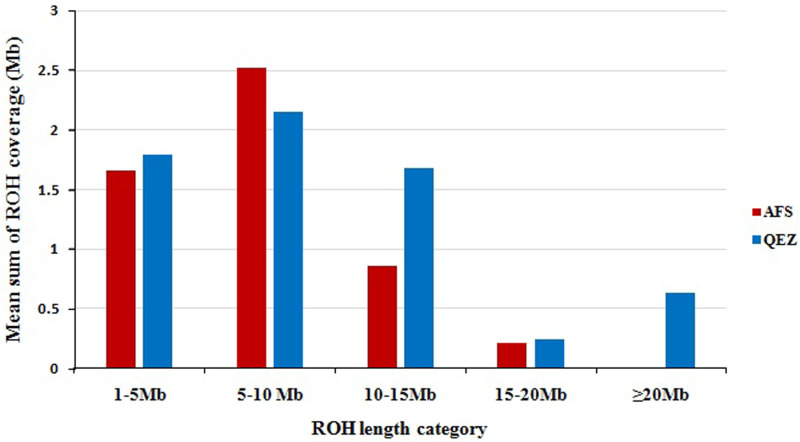
The mean sum of run of homozygosity (ROH) per animal within each ROH length category. For each animal, the ROH lengths within each category were summed and then averaged per population. The red and blue colors are used to show Afshari and Qezel breed animals, respectively.

The majority of the ROH islands observed in this study were short, ranging from 1 to 10 Mb in size (Fig 4). The ROH coverage distribution presented in this study is consistent with previous studies on sheep [19], goats [38], and cattle [43], in which long ROH segments were found less frequently than shorter ones. The higher proportion of ROH segments within the short ROH categories suggests that ancient inbreeding played a larger role [34]. The higher proportion of short ROH segments in Afshari suggests a history of ancient inbreeding, likely resulting from long-term selection for meat production. In contrast, the presence of longer ROH segments in Qezel indicates recent inbreeding, which may reflect more targeted breeding efforts to maintain specific traits, such as milk production.

The mean values of F_ROH_ were 0.005 for Afshari and Qezel, suggesting the low levels of inbreeding in these breeds. These results agree with the findings reported by others (e.g., [44]) showing low levels of F_ROH_ values, ranging from 0.008 (Rasa Aragonesa) to 0.086 (Canaria de Pelo) in Spanish sheep breeds. F_ROH_ value for Jinning Grey Chinese goat breed was reported to be 0.005 [34], which is consistent with our findings. ROH may be helpful to estimate the degree of inbreeding in the utter lack of pedigree records. Due to either incomplete or lacking pedigree information, genomic inbreeding based on ROH provides a more accurate estimate of a person’s autozygosity than pedigree-based inbreeding [3].

### Effective population size (N_e_)

**Fig 5:**
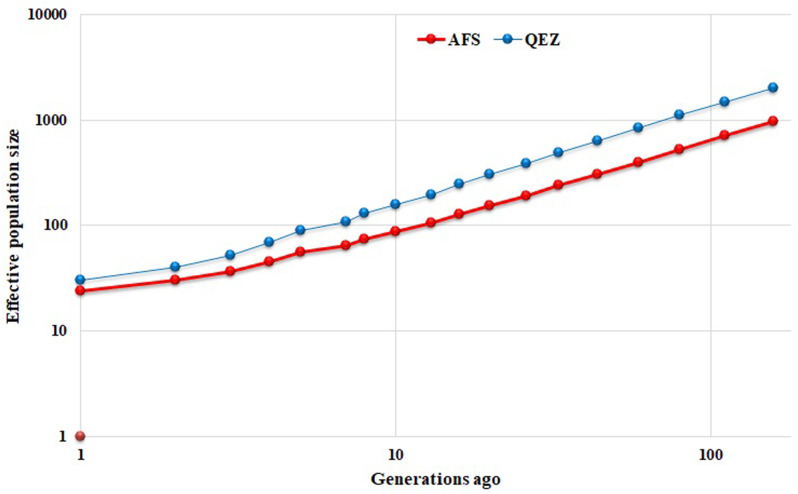
Estimated effective population size (Ne) in different time points in the past (generations ago). The red and blue colors are used to show Afshari and Qezel breed animals, respectively.

The effective population size (N_e_) provides insight into population history and is a general indicator of the danger of genetic erosion, and can be used to track changes in genetic diversity [45]. Fig 5 shows a decreasing trend in estimated N_e_ across generations for both breeds, likely because there are no planned mating strategies in place to preserve genetic diversity in native breeds. Based on our analysis, the Qezel breed had a larger N_e_, than the Afshari breed across all generations. Considering the decreasing trend, the estimated N_e_ in the 5 generations ago ranged from N_e_ = 56 in the Afshari breed to N_e_ = 90 in the Qezel breed.

Burren et al. [46] in a study on seven local Swiss sheep breeds reported decreasing *Ne* as observed in the current study, for instance, *N*_*e*_ of 18, 27, 26, 29, 29, 30, and 31 for Swiss Black-Brown Mountain sheep, Valais Red, Bundner Oberländer, Swiss Mirror, Valais Blacknose, Engadine Red and Swiss White Alpine breeds, respectively in five generations ago. Kim et al. [16] also reported the N_e_ of 130, 45, 21 and 30 for Barki, Rommey, Texel and Corriedale breeds, respectively. Selection and artificial insemination are the main reasons for decreasing the *N*_*e*_ values in different populations [47]. These findings show that Afshari breed, as one of the meat breeds with appropriate daily weight gain and litter size in Iran, has been considered as the desirable breed for rearing among Iranian farmers during recent years. It has been used extensively for fattening and crossbreeding goals in a variety of regions in Iran [48]. The lower N_e_ observed in this breed comparing to Qezel may be due to the high selection intensity that occurred in Afshari sheep breed during the last generations.

While the PCA, Ne, and inbreeding results may appear inconsistent initially, they reflect distinct aspects of genetic diversity and demographic history. The greater spread of Afshari individuals along PC2 suggests higher genetic diversity, likely due to its widespread use for crossbreeding and fattening across Iran. This aligns with the smaller Ne observed in Afshari during last generations, indicating a historically smaller population size and long-term selection for meat production. However, it is important to note that LD-based Ne estimation has limitations, particularly in recent generations, as it relies on the decay of genetic linkages over time and may not fully capture recent demographic changes or selection pressures [29]. Therefore, while Qezel exhibits a higher Ne in recent generations, Afshari may still maintain higher genetic variation due to its extensive crossbreeding, which introduces and preserves genetic diversity.

The higher number of shorter ROHs in Afshari supports evidence of ancient inbreeding, as these segments result from recombination breaking down longer ROHs over many generations. In contrast, Qezel’s larger Ne reflects a historically larger population size and higher genetic diversity, consistent with its role as a dairy breed. However, the lower number of longer ROHs in Qezel suggests recent inbreeding, likely due to targeted breeding for milk production traits. Together, these findings underscore the distinct demographic and selection histories of the two breeds, with Afshari shaped by ancient inbreeding and Qezel influenced by recent inbreeding.

### Identification of selection signatures and gene content

Various methods have been used for identifying the genomic regions under positive selection in common farm animals [1,10,11,16,38,49–51]. In this study, we used three complementary approaches including F_ST_ [31], XP-EHH [14] and hapFLK [15] to assess the genome-wide differences between Iranian indigenous sheep breeds (Afshari and Qezel). Although different approaches may have varying statistical power for detecting selection signatures, but using different methods may boost the accuracy of detection and reduce unknown bias [11,52,53]. The total regions under putative selection identified by the F_ST_, XP-EHH and hapFLK analyses, which overlap with previously reported genes have been listed in Table 1 (a complementary list of regions and their genes can be seen in Supplementary Materials, S4 Table).

**Table 1:** The list of the genes (and their functions) that overlapped with the genomic regions, identified to be under positive selection in Afshari and Qezel sheep breeds, based on each smoothed F_ST_, XP-EHH and hapFLK methods.

OAR*	Position (Mb)	Statistics	Candidate Gene	Gene Function	Reference
1	137.87	XP-EHH	*BTG3*	fetal muscle development milk fat yield	[54] [55]
2	40.20	XP-EHH	*DOCK5*	immune response	[56]
2	174.85-175.12	XP-EHH	*TMEM163*	a putative zinc transporter	[57]
2	209.76-209.79	F_ST_	*MAP2*	nervous system	[58]
2	240.77-240.83	F_ST_	*RUNX3*	immune system	[59]
3	115.56-116.15	F_ST_	*PPP1R12A* MYF6 MYF5	growth and muscular system	[60] [61] [62]
3	162.89-162.90	XP-EHH	*IL23A, STAT2* *TIMELESS* *GLS2*	immune response circadian timing energy metabolism	[63] [64] [65]
3	172.71-172.96	F_ST_	*HCFC2* *HSP90B1* *TXNRD1*	immune system response to environmental stress fatty acids metabolism	[66] [67] [68]
4	71.54	XP-EHH	*NPY*	growth traits	[69]
7	87.45	XP-EHH	*NRXN3*	body fat distribution	[70]
8	7.05	XP-EHH	*BCKDHB* *TTK*	metabolism of amino acids milk protein yield	[71] [55]
8	60.37	XP-EHH	*5S_rRNA*	disease resistance	[5]
9	62.10-62.23	XP-EHH	*TRPS1*	mammary gland morphogenesis and development	[72]
10	41.21	XP-EHH	*PCDH9*	development and function of the nervous and endocrine systems	[73]
10	76.20-76.23	F_ST_	*PCCA*	milk production trait	[74]
12	49.27-49.34	F_ST_	SCNN1D UBE2J2 TNFRSF18 ACAP3	sweat gland development modification of proteins immune system milk protein yield	[17] [75] [76] [55]
20	3.34-4.45	HapFLK	*COL21A1* *BMP5*	Angiogenesis Fecundity	[77] [34]
21	10.74	F_ST_	*DLG2*	nervous system	[78]
26	4.76-5.89	F_ST_ and hapFLK	*ANGPT2*	Reproduction	[79]
26	4.76-5.89	hapFLK	*AGPAT5*	triglyceride biosynthesis	[80]
26	6.14-6.16	HapFLK	*GPM6A*	nervous system	[81]
X	9.92	XP-EHH	*FRMPD4*	nervous system	[82]

*For a complete list of the selected regions and the genes see supplementary S4 Table.

#### F_ST_.

**Fig 6:**
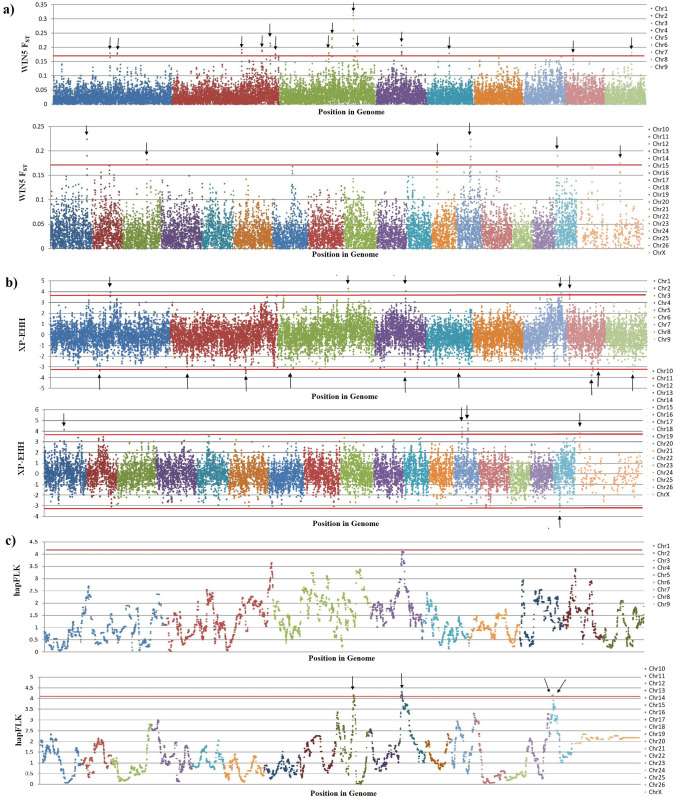
Genomic regions detected to be under selection in Afshari (AFS) and Qezel (QEZ) breeds. (a) Manhattan plot of the windowed fixation index (F_ST_). The red lines represent the 0.1% percentile threshold for F_ST_ > 0.17. (b) Manhattan plot of the cross population extended haplotype homozygosity (XP-EHH) scores. The red lines indicate the 0.1% percentile threshold for -3.19 < XP-EHH < 3.75, and high positive values suggest selection in Afshari and negative values selection in Qezel sheep breeds. (c) Manhattan plot of hapFLK statistics. The red lines are showing the 0.1% percentile threshold for hapFLK > 4.1. In this figure, the SNP positons in the genome (bp) are shown on the X-axis and the windowed F_ST_, XP-EHH scores, and hapFLK values are plotted on Y-axis. For each method, the results of first 9 chromosomes are displayed on the top plot and the results of the remaining chromosomes are shown on the bottom plot.

Fig 6a depicts the plot of windowed F_ST_ against genomic location. Several genomic regions were identified to be under positive selection (20 selectin signatures) on OAR1, 2, 3, 4, 5, 8, 9, 10, 12, 21, 22, 26, and X, that overlapped with several genes within each region of interest (Table 1 and Supplementary Material S4 Table). The regions with the highest F_ST_ values were located on OAR3 between 172893394 and 172830773 kb that overlap with the genes of *HCFC2, HSP90B1*, and *TXNRD1*.

The genes overlapped with the regions under positive selection using F_ST_ approach were involved in meat performance and muscle formation (*MYF5*, *MYF6* and *PPP1R12A*), immune system (*RUNX3*, *TNFRSF18, HSP90B1, HCFC2*), nervous system (*MAP2*, *DLG2*), reproduction performance (*ANGPT2*), milk protein yield (*ACAP3*), sweat gland development (*SCNN1D*) and modification of proteins (*UBE2J2*) (Table 1).

Meat quality is an important economic trait in animal husbandry, as well as a reference index in sheep breeding in Iran. Core genes and signal transduction pathways regulate muscle growth and development [62]. *MYF5* and *MYF6* are regulatory factors belonging to the muscle regulatory factors (MRFs) family, that play crucial rule in muscle differentiation, muscle growth and development [61]. The earliest gene to express during embryonic muscle development is *MYF5*. Pre-adipocytes and neurons are among the other tissues in which this gene is expressed [83], however, its expression in these tissues is less pronounced than in muscle tissues [84]. *MYF6* is primarily involved in the myoblast fusion and differentiation [61]. *MYF5* diversity is reported to be associated with meat quality traits such as intramuscular fat and lean meat content in pigs [85], carcass and body fat weight in cattle [86], growth traits in chickens [87], and live weight and carcass weight diﬀerences in geese [88]. Wang et al. [62] suggested that ovine *MYF5* might be a good genetic marker for getting more lean meat from sheep.

*PPP1R12A* (*MYPT1*) gene encodes a regulatory subunit of myosin light chain phosphatase, which is crucial for smooth muscle contraction. Mutation of this gene boosts myosin phosphorylation, contractility, and blood pressure. *PPP1R12A* is implicated in the modulating of vascular smooth muscle contractility [60]. *PPP1R12A* has been identified to be under selection in the comparison of Ethiopian sheep populations and is known to be associated with altitude adaptation [89] and hypoxia increased phosphorylation of *PPP1R12A* gene [90].

The development of immune system cells is regulated by transcription that is mediated by *RUNX3*. Innate lymphoid cells (ILCs) of the *ILC1* and *ILC3* lineages, which reside in the mucosa and participate in the response to external pathogens, cannot develop without *RUNX3*. By promoting the expression of the RORC (RORgamma) gene, which codes for the nuclear retinoid-related orphan receptor-gamma, *RUNX3* contributes to the growth of the *ILC1* and *ILC3* lineages [59]. This gene controls the transcription of integrin genes, which are necessary for leukocyte migration during immunological and inflammatory responses [91].

Thermal stress refers to either heat or cold stress. These changes demand the adaptation of animals to harsh environments. Heat Shock Proteins (*HSPs*) are a family of the evolutionarily conserved proteins that are induced in living cells in response to biological stress [92]. Cellular tolerance to heat stress is regulated by the *HSPs*, which act as molecular chaperons to prevent abnormal protein folding and aggregation. These proteins enhance the cell’s capacity and tolerance to withstand injury, oxidative stress and high temperatures [67]. Salama et al. [93] reported an increased *HSP90B1* expression in milk cells of dairy goats under heat stress.

The primary function of sweat glands in maintaining homeostasis is stabilizing body temperature. Various genes and pathways play roles at different stages of sweat gland development and thermal sweating. Wnt signaling is essential for the induction of sweat glands in epidermal progenitor cells [17]. The *ENaC* gene, which also called positively selected *SCNN1D* gene, is found in sweat glands and helps body sodium absorbtion [94]. Taye et al. [17] reported a regions to be under selection in the *SCNN1D* gene located on BTA16 in African cattle which is consistent with finding of our study.

Weikard et al. [95] investigated tissue-specific regulatory mechanisms required for milk production in cows. The comparison of gene expression patterns revealed that *PCCA* gene has been participated with metabolic adaptation to divergent milk production performance. This gene has also been found to be under selection in Russian cattle [74].

Thioredoxin reductase (*TrxR*) in mammals is a selenoprotein with three isoenzymes (*TrxR1*, *TrxR2*, and *TrxR3*). The Trx/TrxR system plays a crucial role in adipose tissue physiology, carbohydrate metabolism, insulin production and sensitivity, blood pressure regulation, and inflammation [68]. The founding of our study are supported by Urbinati et al. [96], who used methodologies based on the extended haplotype homozygosity and discovered evidence of selection in the region on BTA5 including *Trxr1* (*TXNRD1*) which is involved in fatty acids metabolism in Canchim beef cattle.

#### XP-EHH.

The XP-EHH statistics identified a total of 9 and 10 genomic regions subjected to positive selection in Afshari and Qezel sheep, respectively (Fig 6b). The top XP-EHH signals within genic regions in the two breeds are summarized in Table 1 (see more details in Supplementary Material S4 Table). These regions overlapped with some important genes associated with growth traits and muscular development (*NPY, BTG3*), energy metabolism (*GLS2*), immune response (*IL23A, STAT2, DOCK5*), disease resistance traits (*5S_rRNA*), nerves system *(FRMPD4, PCDH9*) and body fat distribution (*NRXN3*) (Table 1).

The most important economic traits of livestock, especially for meat production, are growth and body weight traits. Meat-type animals can be distinguished by their body weight, which can be measured at birth or at various points of life stages. One of the most potent orexigenic factors, Neuropeptide Y (*NPY*), influences various behaviors and physiological functions, with its most prominent role being the stimulation of appetite. Zhang et al. [69] found that *NPY* gene variants significantly affect body length and chest girth aged 6, 12 and 18 months in Chinese indigenous cattle, suggesting it controls feeding behavior and energy homeostasis, as revealed by their study on Nanyang cattle. Studies in humans also suggested that *NPY* polymorphisms influence obesity in males and was associated with birth weight, body fat patterning and increased leptin levels [97]. The BTG Anti-Proliferation Factor 3 (*BTG3*) gene, belongs to a member of the BTG/Tob family, which has structurally associated with proteins that appear to have anti-proliferative properties. Previous studies showed that this gene was associated with fetal muscle development [98]. Additionally, Suchocki et al. [55] discovered that the *BTG3* gene is crucial for milk fat yield in Polish Holstein dairy cattle through gene networks analysis.

Glutaminase 2 (*GLS2*) regulates energy metabolism by converting glutamine to glutamate, which increases mitochondrial ATP production [99]. This gene has been previously reported as being under positive selection due to its key role in ATP generation [65].

It is known that several cytokines are signiﬁcantly involved in immune system of sheep infected with Haemonchus contortus [100]. Onzima et al. [2] reported the selection signatures in Ugandan goat breeds and identified *IL23A* gene that is associated with disease resistance. The identiﬁcation of cytokines like *IL23A* in our study may be associated with gastrointestinal parasite resistance in these breeds, suggesting that immunity genes are key targets of natural selection in Iranian sheep breeds due to local pathogen and parasite challenges [101,102].

Numerous potential genes have been identified in the genomic regions linked to inflammation, host defense mechanism and diseases resistance. One of the most important genes is *5S_rRNA*, which is a gene identified in several overlapped regions (OAR2 and OAR8). Abied et al. [5] in a study on five Chinese native sheep breeds adapted to extremely dry and humid environments showed that the genomic regions detected on OAR2 and OAR6 include *5S_rRNA* gene associated with disease resistance traits.

#### hapFLK.

Fig 6c depicts the results of the haplotype-based differentiation with hapFLK in Afshari and Qezel sheep breeds. The hapFLK analysis resulted in four putative selection signature regions on OAR 18, 20 and 26. These regions overlapped with the genes that are associated with angiogenesis (*ANGPT2, COL21A1*), fecundity and growth (*BMP5*), and nerves system (*GPM6A*) (Table 1).

To support fetal growth and development during pregnancy, placental vascular development (angiogenesis) occurs concurrently with the expansion of uterine and placental tissues. The placenta is a vital organ for maternal-fetal exchange, rapidly growth, requires high metabolic demand, and relies on a dynamic angiogenic process throughout pregnancy [79]. Angiogenesis is a process that is essential for growth and development of all tissues, including the placenta. *ANGPTs* [103] and *COL21A1* [77] are recognized as the major regulators of vasculogenesis and angiogenesis in placenta. In the development of the in-utero feto-placental circulation system, Dunk et al. [104] showed the roles of *ANGPTs* (*ANGPT1*/*ANGPT2*) in vascular endothelium as regulators of trophoblast behavior.

*BMP5* (Bone Morphogenetic Protein 5), located on chromosome 23, plays a crucial role in the regulation of growth and ovulation in animals [105]. *BMP5* belongs to the BMP subfamily and plays a significant role in ovarian folliculogenesis [106]. Islam et al. [34] showed that *BMP5* gene involved in goat fecundity. The *BMP5* gene has been also suggested to be a potential candidate gene for fatness traits. Considering the chromosome position of the *BMP5* gene that is located within QTL region for fatness, and its function on skeletal and muscle development, *BMP5* gene may be potentially related with fecundity and growth [107].

Studies done retrospectively on humans and on animals have shown that early exposure to negative events, such as prenatal stress, can change adult behavior. Stress during pregnancy has a significant impact on how the fetal brain develops and how the adult brain functions. The PLP/DM20 (proteolipid protein) family member glycoprotein M6A (*GPM6A*) is a neuronal transmembrane protein that associates with lipid rafts rich in cholesterol and facilitates the formation of filopodia. Prenatal stress alters the expression of the glycoprotein *GPM6A* gene and causes epigenetic modifications in rat offspring brain [81].

### Enrichment analysis

Finally, the genes located near each region of interest were collected for all methods and pooled for functional analysis. We then examined the Gene Ontology (GO) terms linked to these genes to check for evidence of functional enrichment. This revealed enrichment for GO terms associated with growth (GO:0040007), skeletal system development (GO:0001501), developmental process (GO:0032502), immune system process (GO:0002376) and nervous system development (GO:0007399) (Table 2). Likely candidate genes identified with the putative selection signatures included those involved in muscle formation and body fat distribution (*BTG3*, *PPP1R12A*, *MYF5*, *MYF6*, *NPY*, *NRXN3* and *BMP5*), immune (*DOCK5*, *RUNX3*, *IL23A*, *STAT2*, *HCFC2*, *TNFRSF18* and *5S*-rRNA), fecundity (*BMP5*) and nerves system (*MAP2*, *PCDH2*, *DLG2*, *GPM6A* and *FRMPD4*) traits.

**Table 2:** Enrichment analysis of gene ontology (GO) terms for all the genes located in the regions under putative selection in two Iranian sheep breeds.

GO term	Description	P-value		
**F**_**ST**_	**XP-EHH**	**HapFLK**
GO:0009605	Response to external stimulus	3.2E-1	–	–
GO:0000003	Reproduction	9.8E-1	–	–
GO:0006807	Nitrogen compound metabolic process	9.1E-1	–	–
GO:0010467	Gene expression	7.6E-1	–	–
GO:0019538	Protein metabolic process	9.0E-1	–	–
GO:0002376	Immune system process	9.6E-1	6.1E-1	–
GO:0040007	Growth	–	3.6E-2	–
GO:0032502	Developmental process	–	1.3E-1	–
GO:0006915	Apoptotic process	–	2.0E-1	–
GO:0048856	Anatomical structure development	–	2.2E-1	–
GO:0009987	Cellular process	–	9.8E-1	3.9E-1
GO:0048731	System development	–	–	3.2E-2
GO:0007399	Nervous system development	–	–	6.4E-1
GO:0050896	Response to stimulus	–	–	1.8E-1
GO:0001501	Skeletal system, development		–	2.2E-1

In general, as described previously, two breeds assessed in the current study namely Afshari and Qezel located in a very close geographical area with very similar morphological characteristics, while reared for different production traits consisting fattening in Afshari and both wool and milk production in Qezel. Both breeds are well adapted to the dry and cold local climate and disease resistance in the north western of Iran. Since body weight at various ages and daily growth rates are crucial economic traits in Afshari sheep, the results of our study revealed different candidate genes associated with growth and muscle formation (e.g., *NPY*, *MYF5* and *PPP1R12A*), body fat distribution (*NRXN3*) and energy metabolisms (e.g., *GLS2*). Also, the Lighvan cheese, a traditional and highly valued kind of Iranian cheese, is basically made from Qezel sheep raw milk. Several candidate genes were identified, which are associated with mammary gland development (e.g., *TRPS1*) and milk traits (e.g., *PCCA*, *ACAP3*, *TTK* and *BTG3*). Furthermore, our results showed that production-related pathways and environmental adaptability mechanisms, such as immune response (e.g., *IL23A, STAT2* and *DOCK5*), sweating and sweat gland development (e.g., *SCNN1D*), reproduction (e.g., *ANGPT2*), fecundity (e.g., *BMP5*) and nervous system (e.g., *DLG2*, *PCDH9* and *FRMPD4*) may be under selection in Iranian sheep breeds.

Some of these genes were briefly described functionally in this paper, however, additional studies with a higher sample size or an independent population will be required to confirm our results. Furthermore, even though plausible candidate genes were identified in several regions of interest harboring selection signatures in the present study, however, many non-coding regions and uncharacterized genes also lived within these regions that cannot be dismissed. As these uncharacterized genes and regions may contribute to phenotypic variability in performance traits, or traits linked to disease resistance or environmental adaptations, more annotation and research into their functional properties are required. Also while the ROH and selection signature analyses were conducted independently, they provide complementary insights into the genomic diversity and evolutionary history of the Afshari and Qezel breeds. ROH analysis revealed patterns of autozygosity that reflect recent and historical inbreeding, while selection signature analysis identified genomic regions under positive selection, likely due to adaptation or artificial selection. Although we did not perform a formal comparison of ROH islands and selection signatures, it is possible that historical inbreeding and selection pressures have interacted to shape the genomic architecture of these breeds. Future studies could explore these interactions in greater detail by integrating ROH and selection signature analyses.

## Conclusion

The pattern of ROH, N_e_ and selection signatures were investigated in two main Iranian sheep breeds in this study including Afshari and Qezel using genome-wide SNP data. Our findings revealed differences in ROH length patterns and burden between the breeds, with Afshari sheep exhibiting a higher number of ROH compared to Qezel sheep. Furthermore, the results showed that N_e_ has declined strongly in Iranian sheep breeds, particularly in Afshari sheep breeds during recent years. Therefore, it is crucial to design appropriate conservation programs to preserve the remaining purebred animals of these native sheep breeds. The study of selection signatures in these breeds revealed some novel selection signatures as well as some other genomic regions under selection, that overlapped with the genes associated with growth, energy metabolisms, reproduction, immune, nervous system and milk production traits. These findings are useful for genomic studies, genetic improvement programs, and sheep breeding.

## Supporting information

S1 Table Summary table for data cleaning in Afshari and Qezel sheep breeds used for ROH and Ne calculations. (PDF)

S2 Table Summary table for data cleaning in Afshari-Qezel sheep breeds used for F_ST_ and hapFLK statistics. (PDF)

S3 Table Summary table for data cleaning in in Afshari-Qezel sheep breeds used for XP-EHH statistic. (PDF)

S4 Table The genomic regions under selections and the list of the genes located at the position based on F_ST_, XP-EHH and hapFLK approaches. (PDF)
