# Supplementary material for: Genomic insights into runs of homozygosity, effective population size and selection signatures in Iranian meat and dairy sheep breeds
Source: PLoS One. 2025 Jun 11;20(6):e0323328. doi: 10.1371/journal.pone.0323328 (PMC12157092; doi:10.1371/journal.pone.0323328)
Supplement: S1 Table — (PDF) [file pone.0323328.s001.pdf]

Quality control in data set:	Afshari	Qezel
Number of Animals	41	35
Excluding Animals with 95% Call rate	4	-
Number of SNPs	49017	49017
Excluding SNPs with MAF≤5% *	4659	3246
Excluding SNPs with deviation from HWE (<0.000001)	1	728
Excluding SNPs with unknown chromosomal position	205	219
Remained SNPs	44152	44824

* MAF: minor allele frequency; HW: Hardy-Weinberg equilibrium
