# Supplementary material for: Genomic insights into runs of homozygosity, effective population size and selection signatures in Iranian meat and dairy sheep breeds
Source: PLoS One. 2025 Jun 11;20(6):e0323328. doi: 10.1371/journal.pone.0323328 (PMC12157092; doi:10.1371/journal.pone.0323328)
Supplement: S2 Table — (PDF) [file pone.0323328.s002.pdf]

Quality control in Afshari-Qezel data set	
Number of Animals	76 (41 Afshari samples and 35 Qezel samples)
Excluding Animals with 95% Call rate	4 Afshari samples
Number of SNPs	49017
Excluding SNPs with MAF ≤2% over all animals *	1365
Excluding SNPs with deviation from HWE (<0.000001)	788 (19 Afshari samples and 769 Qezel samples)
Excluding SNPs with unknown chromosomal position	215
Remained SNPs	46649

* MAF: minor allele frequency; HW: Hardy-Weinberg equilibrium
