# Supplementary material for: Genomic insights into runs of homozygosity, effective population size and selection signatures in Iranian meat and dairy sheep breeds
Source: PLoS One. 2025 Jun 11;20(6):e0323328. doi: 10.1371/journal.pone.0323328 (PMC12157092; doi:10.1371/journal.pone.0323328)
Supplement: S3 Table — (PDF) [file pone.0323328.s003.pdf]

Quality control in Afshari-Qezel data set	
Number of Animals	76 (41 Afshari samples and 35 Qezel samples)
Excluding Animals with 95% Call rate	4 Afshari samples
Number of SNPs with information of ancestral alleles	34213
Excluding SNPs with MAF≤2% over all animals	977
Excluding SNPs with deviation from HWE (<0.000001)	581 (7 Afshari samples and 574 Qezel samples)
Excluding SNPs with unknown chromosomal position	150
Remained SNPs	32505
