# Supplementary material for: Genomic insights into runs of homozygosity, effective population size and selection signatures in Iranian meat and dairy sheep breeds
Source: PLoS One. 2025 Jun 11;20(6):e0323328. doi: 10.1371/journal.pone.0323328 (PMC12157092; doi:10.1371/journal.pone.0323328)
Supplement: S4 Table — (PDF) [file pone.0323328.s004.pdf]

OAR	Position (Mb)	Statistic	Genes
2	162.26-162.32	F_{ST}	*U6*
	40.20	XP-EHH	*5S_Rrna*
	174.85-175.12	XP-EHH	*ACMSD, RF00026*
	228.78-228.84	F_{ST}	*AGFG1, SLC19A3*
3	115.56-116.15	F_{ST}	*OTOGL*
	182.14	F_{ST}	*BICD1, FGD4*
4	60.92-60.95	F_{ST}	*KIAA0895, EEPD1*
	69.15	XP-EHH	*SKAP2, HOXA1*
5	52.45	F_{ST}	*YIPF5*
8	16.97	F_{ST}	*TBC1D32*
	57.01-57.03	XP-EHH	*ENPP1*
	60.37	XP-EHH	*MYB*
12	49.27-49.34	F_{ST}	*CPTP, CPSF3L, PUSL1,FAM132A, B3GALT, SDF4*
18	35.16-35.37	hapFLK	*NOVA1*
22	16.47-16.56	XP-EHH	*TCTN3*
	28.78-28.90	F_{ST} and XP-EHH	*XPNPEP1*
26	4.76-5.89	hapFLK	*XKR5*
	13.40-13.48	XP-EHH	*ENPP6*
